# Postoperative fibrinogen‐to‐albumin ratio acting as an indicator of futile recanalization in patients with successful thrombectomy

**DOI:** 10.1002/brb3.3301

**Published:** 2023-11-01

**Authors:** Tao Tang, Di Li, Tie‐Ping Fan, Lin‐Jia Guo, Xiao‐Yan Lan, Cong‐Jie Bi, Johannes Boltze, Aline M. Thomas, Xu‐Sheng Zhao, Ming Mo, Man‐Hong Zhao, Xun‐Ming Ji, Shen Li

**Affiliations:** ^1^ Department of Neurology and Psychiatry Beijing Shijitan Hospital, Capital Medical University Beijing China; ^2^ Department of Neurointervention Central Hospital of Dalian University of Technology Dalian China; ^3^ Department of Anesthesiology Central Hospital of Dalian University of Technology Dalian China; ^4^ School of Life Sciences University of Warwick Coventry UK; ^5^ The Russell H. Morgan Department of Radiology and Radiological Sciences The Johns Hopkins University School of Medicine Baltimore Maryland USA; ^6^ Beijing Institute of Brain Disorders Capital Medical University Beijing China

**Keywords:** fibrinogen‐to‐albumin ratio, futile recanalization, stroke, thrombectomy

## Abstract

**Background:**

Timely recognition of futile recanalization might enable a prompter response and thus improve outcomes in patients receiving successful thrombectomy. This study aims to evaluate whether postoperative fibrinogen‐to‐albumin ratio (FAR) could act as an indicator of futile recanalization.

**Methods:**

This is a single‐center, retrospective analysis of patients with acute anterior circulation large‐vessel occlusion and successful thrombectomy between May 2019 and June 2022. FAR was defined as postoperative blood levels of fibrinogen divided by those of albumin, and dichotomized into high and low levels based on the Youden index. Futile recanalization was defined as patients achieving a successful recanalization with a modified Rankin Scale score of 3–6 at 90 days. Multivariable logistic regression was used to assess the association of FAR with futile recanalization.

**Results:**

A total of 255 patients were enrolled, amongst which 87 patients (34.1%) had high postoperative FAR. Futile recanalization was more prevalent among patients with high FAR compared to those with low FAR (74.7% vs. 53.0%, *p* = .001). After adjusting for potential confounders, high postoperative FAR was found to independently correspond with the occurrence of futile recanalization (adjusted OR 2.40, 95%CI 1.18–4.87, *p* = .015). This association was consistently observed regardless of prior antithrombotic therapy, treatment of intravenous thrombolysis, occlusion site, time from symptom onset to groin puncture, and reperfusion status.

**Conclusion:**

Our findings support high postoperative FAR serving as an indicator of futile recanalization in patients with anterior circulation large‐vessel occlusion and successful thrombectomy.

## Graphical abstract

Timely recognition of futile recanalization might enable a prompter response and thus improve outcome in patients receiving successful thrombectomy. The fibrinogen‐to‐albumin ratio is a valuable serological marker that may reflect information on both blood hemorheology and inflammation. Futile recanalization was more prevalent among patients with high postoperative fibrinogen‐to‐albumin ratio. High postoperative fibrinogen‐to‐albumin ratio could serve as an indicator of futile recanalization.

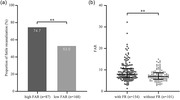

## 1 INTRODUCTION

Stroke poses a major health and social economic burden worldwide (Collaborators, 2021). In China, it is the leading cause of death and acquired adult disability, and its prevalence continues to increase (Tu & Wang, 2023; Tu et al., 2023). Mechanical thrombectomy has now emerged as the standard treatment for acute ischemic stroke with proximal intracranial large‐vessel occlusion (Goyal et al., 2016). However, approximately half of treated patients do not achieve functional independence despite receiving a successful recanalization (Kim et al., 2021). This paradoxical phenomenon has been termed futile recanalization (Deng et al., 2022). Timely recognition of futile reperfusion may enable the use of alternative and/or adjuvant treatments such as glycoprotein IIb/IIIa receptor inhibitor infusion for intracranial atherosclerotic disease (Sang et al., 2023) and early anticoagulation for patients with atrial fibrillation (Xu et al., 2023), and thus improve patient outcomes. Therefore, identifying indicators of futile recanalization has gained attention in recent years (Feng et al., 2022).

Having an older age, a history of hypertension, a higher admission systolic blood pressure, a higher National Institutes of Health Stroke Scale (NIHSS) score, or a delayed intervention is associated with futile recanalization (Nie et al., 2023; Wang & Xiong, 2023). In addition, blood biomarkers may also serve as a practical tool to indicate the pathophysiological status of the patient and identify therapeutic targets of futile recanalization (Zang et al., 2020). The fibrinogen‐to‐albumin ratio (FAR) has been suggested as a novel marker of disease severity in prothrombotic conditions (Roth et al., 2021). Fibrinogen is the most abundant clotting factor in the body that is associated with fibrin formation and platelet aggregation. It is also an acute‐phase reactant, reflecting a state of systemic inflammation (Acharya et al., 2020). Albumin is a plasma protein that has both antiplatelet and anti‐inflammatory properties (Acharya et al., 2020; Ruan et al., 2021). Taken together, FAR has been described as a valuable serological marker that may reflect information on blood hemorheology and inflammation (Xiao et al., 2019). A previous study found that high FAR could predict the occurrence of no‐reflow phenomenon in patients with myocardial infarction undergoing primary percutaneous coronary intervention (Zhao et al., 2019). As no‐reflow phenomenon and inflammatory response are proposed underlying mechanisms of futile recanalization (Deng et al., 2022; Wang & Xiong, 2023), FAR might also be a potential indicator of futile recanalization in acute ischemic stroke patients. This study aims to evaluate whether postoperative FAR could act as an indicator of futile recanalization in patients with successful thrombectomy.

## 2 METHODS

### 2.1 Study design and subjects

Between May 2019 and June 2022, patients undergoing mechanical thrombectomy for acute ischemic stroke within 24 h of symptom onset at Central Hospital of Dalian University of Technology were recruited in this retrospective study. Patients were managed according to current guidelines (Powers et al., 2019). Patients were included if they (1) had a proximal anterior circulation occlusion (intracranial internal carotid artery, middle cerebral artery (M1 segment),or both); (2) were older than 18 years; (3) had a pre‐stroke modified Rankin Scale (mRS) score ≤2; (4) had a successful recanalization, defined by a final modified Thrombolysis in Cerebral Infarction (mTICI) score of 2b or 3; (5) had both blood fibrinogen and albumin levels tested within 24 h after thrombectomy; and (6) had a functional outcome assessment using mRS at 90 days.

The Central Hospital of Dalian University of Technology Ethics Committee approved this study (2019‐004‐11). Each patient gave written, informed consent on admission for all diagnostic and therapeutic procedures and was informed that nonpersonal information may be used for clinical investigations. All relevant information was obtained from the clinical database of the Central Hospital of Dalian University of Technology retrospectively without reinforming the patients. Only anonymized data were used. As the patients’ privacy was not violated, the waiver of post hoc written informed consent for using the data for scientific purposes was approved by the Ethics Committee. The study was conducted according to the principles expressed in the Declaration of Helsinki.

### 2.2 Data collection

The following data were collected: age, sex, pre‐stroke mRS score, medical history including main vascular risk factors (hypertension, diabetes mellitus, previous ischemic stroke or transient ischemic attack, atrial fibrillation, prior antithrombotic therapy, and current smoking), systolic and diastolic blood pressure at admission, baseline NIHSS score, baseline Alberta Stroke Program Early CT Score, blood glucose at admission, treatment with intravenous thrombolysis, occlusion site determined by digital subtraction angiography, collateral status, anesthesia type, time from symptom onset to groin puncture, time from onset to reperfusion, device‐pass number, reperfusion status, stroke subtype according to the Trial of Org 10172 in Acute Stroke Treatment classification (Adams et al., 1993), and blood fibrinogen and albumin levels of fasting venous blood samples collected at the first postoperative morning.

FAR was defined as the postoperative fibrinogen level (mg/dL) divided by the albumin level (g/L). Collateral status was evaluated at the time of the pre‐thrombectomy angiogram, which was dichotomized into good (grade 3–4) and poor (grade 0–2) collaterals according to the American Society of Interventional and Therapeutic Neuroradiology/Society of Interventional Radiology collateral flow grading system (Anadani et al., 2022). Reperfusion status was evaluated using the mTICI score (Zaidat et al., 2013). Imaging variables were analyzed by two experienced neurointerventionalists (>10 years of experience) blinded to patients’ information.

### 2.3 Outcome

Futile recanalization was defined as patients achieving a successful recanalization with an mRS of 3–6 90 days after thrombectomy (Yang et al., 2023), which was assessed by stroke neurologists during the clinical follow‐up visits or via standardized telephone interviews with the patient or their caregivers.

### 2.4 Statistical analysis

Shapiro–Wilk test was used to test data distribution. Categorical variables were expressed as frequencies and percentages. Continuous variables were expressed as mean ± standard deviation, or median (interquartile range) when non‐normally distributed. Baseline characteristics were compared using Student *t* test/Mann–Whitney *U* test, or *χ* ^2^ test/Fisher's exact test, as appropriate, according to the type of variables and their distribution.

Postoperative FAR was dichotomized into high and low levels based on the Youden index. The association between high postoperative FAR and futile recanalization was then evaluated by a multivariable logistic regression model. To select other potential confounders and maximize sensitivity, we first added variables with *p* < .10 in the univariate analysis to the model. Then, we excluded variables one‐by‐one from the model by backward elimination. The elimination criterion was *p* ≥ .10. Multiplicative interaction analyses were performed to evaluate the heterogeneity of the association of high postoperative FAR with futile recanalization between the subgroups of each category, which included a prior history of receiving antithrombotic therapy (yes vs. no), treatment of intravenous thrombolysis (with vs. without), occlusion site (internal carotid artery vs. middle cerebral artery), time from symptom onset to groin puncture (≤360 vs. >360 min), and reperfusion status (mTICI 2b vs. mTICI 3). To minimize the bias resulting from variables with missing data, multiple imputation was performed using other variables listed in Table 1 with a predictive mean matching method (chained equations with 20 iterations) under the missing at random assumption. Estimates obtained in imputed datasets were combined using Rubin's rules (Austin et al., 2021). Finally, we conducted a sensitivity analysis to examine the potential impact of missing data. We reported the adjusted odds ratios (aOR) for multivariable analyses with a 95% confidence interval (CI). All tests were 2‐tailed with a significance level of 0.05. All analyses were performed with STATA software (StataCorp LLC).

**TABLE 1:** Baseline characteristics of all patients and according to fibrinogen‐to‐albumin ratio (FAR) levels.

Variables	All patients (*N* = 255)	High FAR (*N* = 87)	Low FAR (*N* = 168)	*p* Value
Age (years)	70 (62–76)	70 (65–78)	69 (62–76)	.143
Sex, female, *n* (%)	87 (34.1)	30 (34.5)	57 (33.9)	.929
Pre‐stroke mRS ≥ 1, *n* (%)	13 (5.1)	6 (6.9)	7 (4.2)	.347
Medical history, *n* (%)				
Hypertension	141 (55.3)	51 (58.6)	90 (53.6)	.442
Diabetes mellitus	65 (25.5)	31 (35.6)	34 (20.2)	**.007**
Ischemic stroke/TIA	30 (11.8)	13 (14.9)	17 (10.1)	.257
Atrial fibrillation	105 (41.2)	38 (43.7)	67 (39.9)	.559
Antithrombotic therapy	35 (13.7)	14 (16.1)	21 (12.5)	.429
Current smoking	97 (38.0)	31 (35.6)	66 (39.3)	.569
Current stroke event				
Systolic blood pressure (mmHg)	144.5 ± 27.9	145.9 ± 30.1	143.7 ± 26.7	.551
Diastolic blood pressure (mmHg)	79.8 ± 15.4	80.4 ± 16.2	79.5 ± 15.1	.671
Baseline NIHSS score	18 (14–22)	18 (14–23)	17 (14–21)	.191
ASPECTS	8 (7–10)	8 (7–10)	9 (7–10)	.319
Admission glucose (mmol/L)^a^	7.33 (6.42–9.25)	7.38 (6.61–9.85)	7.33 (6.40–9.00)	.405
Intravenous thrombolysis, *n* (%)	113 (44.3)	22 (25.3)	91 (54.2)	**<.001**
Occlusion site, *n* (%)				.734
Intracranial ICA	121 (47.5)	40 (46.0)	81 (48.2)	
M1	134 (52.5)	47 (54.0)	87 (51.8)	
Poor collaterals, *n* (%)	191 (74.9)	70 (80.5)	121 (72.0)	.141
Anesthesia, *n* (%)				.279
General anesthesia	14 (5.5)	4 (4.6)	10 (6.0)	
Local anesthesia	123 (48.2)	48 (55.2)	75 (44.6)	
Conscious sedation	118 (46.3)	35 (40.2)	83 (49.4)	
Onset to groin puncture time (min)	270 (185–365)	280 (215–415)	260 (175–347)	.050
Onset to reperfusion time (min)	326 (253–420)	350 (269–475)	315 (240–402)	**.042**
Device‐pass number	2 (1–3)	1 (1–3)	2 (1–3)	.264
Reperfusion status, *n* (%)				.382
mTICI 2b	131 (51.4)	48 (55.2)	83 (49.4)	
mTICI 3	124 (48.6)	39 (44.8)	85 (50.6)	
Stroke subtype, *n* (%)				.651
Cardioembolism	132 (51.8)	45 (51.7)	87 (51.8)	
Large‐artery atherosclerosis	117 (45.9)	41 (47.1)	76 (45.2)	
Others	6 (2.3)	1 (1.2)	5 (3.0)	
Postoperative FAR	7.55 (5.98–10.20)	11.89 (10.03–14.17)	6.39 (5.37–7.53)	**<.001**

*Note*: Data are expressed as median (interquartile range), mean ± standard deviation, or *n* (%). *p* Values of <.05 are shown in bold.

Abbreviations: ASPECTS, Alberta Stroke Program Early CT Score; ICA, internal carotid artery; M1, the first segment of middle cerebral artery; mRS, modified Rankin Scale; mTICI, modified Thrombolysis in Cerebral Infarction.; NIHSS, National Institutes of Health Stroke Scale; TIA, transient ischemic attack.

^a^ 31 missing data.

## 3 RESULTS

### 3.1 Baseline characteristics

**FIGURE 1:**
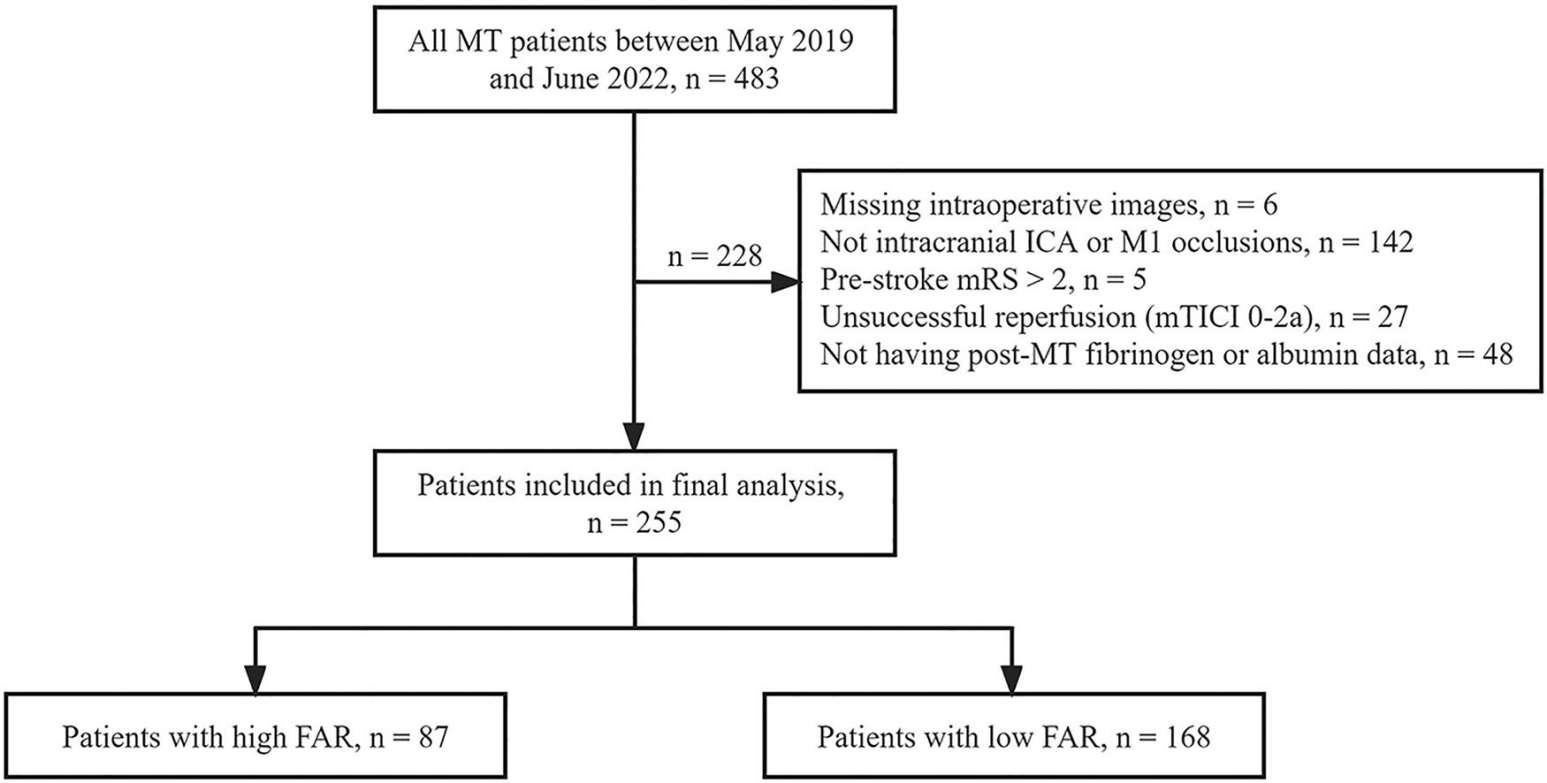
Flowchart illustrating the study inclusion/exclusion and grouping process. MT, mechanical thrombectomy; ICA, internal carotid artery; M1, the first segment of middle cerebral artery; mRS, modified Rankin Scale; mTICI, modified Thrombolysis in Cerebral Infarction; FAR, fibrinogen‐to‐albumin ratio.

The patient selection process is illustrated in Figure 1. A total of 255 patients were included in this study. The median age was 70 (62–76) years and 87 patients (34.1%) were female. The patients had a median NIHSS score of 18 (14−22). Postoperative FAR was dichotomized into high (≥8.94) and low (<8.94) levels based on the Youden index. Table 1 summarizes the baseline characteristics of all patients and according to postoperative FAR levels. Amongst the included variables, only blood glucose at admission had missing data (31, 12.2%). In total, 87 patients (34.1%) had high postoperative FAR. Compared to patients with low FAR, those with high FAR had more diabetes mellitus (35.6% vs. 20.2%, *p* = .007), less treatment of intravenous thrombolysis (25.3% vs. 54.2%, *p* = .001), and longer onset to reperfusion time (350 vs. 315 min, *p* = .042).

### 3.2 Outcome

**FIGURE 2:**
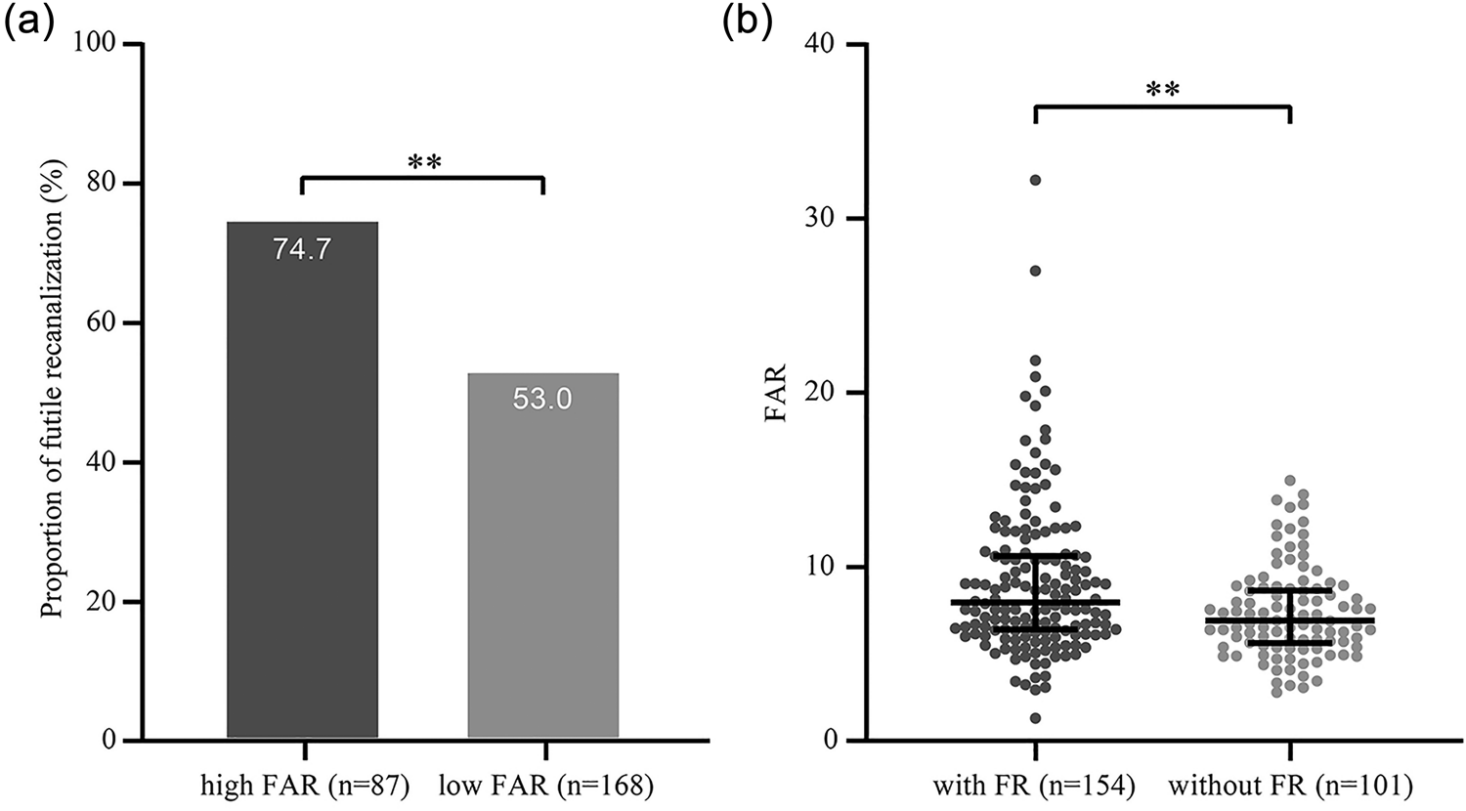
The relationship between postoperative fibrinogen‐to‐albumin ratio (FAR) and the incidence of futile recanalization. (a) Futile recanalization was more prevalent among patients with high FAR compared to those with low FAR (74.7% vs. 53.0%, *p* = .001). (b) The postoperative FAR was higher in patients with futile recanalization than those without (7.96 [6.25–10.81] vs. 6.93 [5.48–8.80], *p* = .002). **p < .01.

**FIGURE 3:**
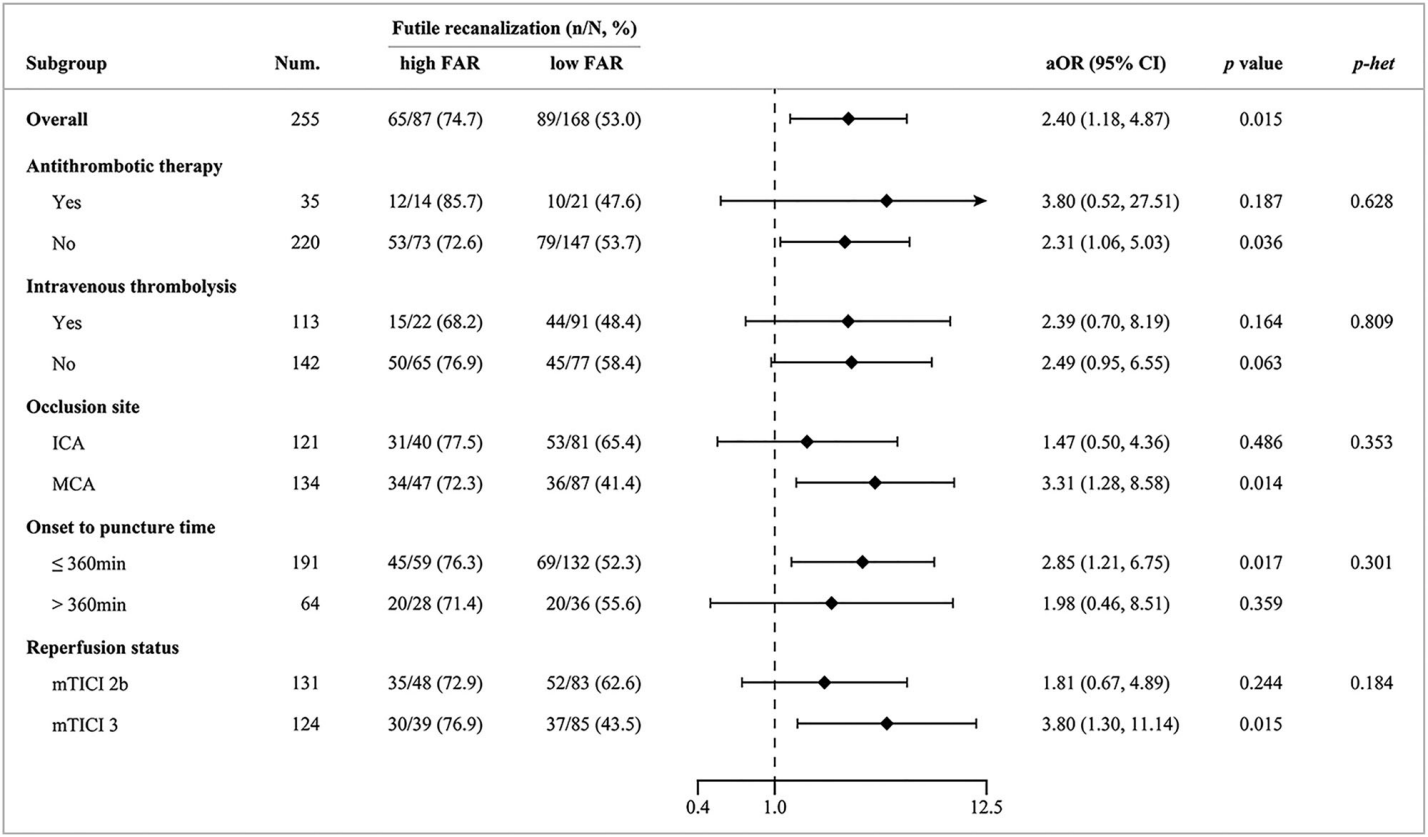
Influence of high postoperative fibrinogen‐to‐albumin ratio (FAR) on futile recanalization in all patients and based on prior antithrombotic therapy, treatment of intravenous thrombolysis, occlusion site, time from symptom onset to groin puncture, and reperfusion status. *Note*: Adjusted odds ratios (aOR) was adjusted for age, baseline National Institutes of Health Stroke Scale (NIHSS) score, blood glucose levels at admission, occlusion site, and collateral status. CI, confidence interval; ICA, internal carotid artery; MCA, middle cerebral artery; mTICI, modified Thrombolysis in Cerebral Infarction.

Futile recanalization was more prevalent among patients with high FAR compared to those with low FAR (74.7% vs. 53.0%, *p* = .001), and the postoperative FAR was higher in patients with futile recanalization than those without (7.96 vs. 6.93, *p* = .002; Figure 2). Multivariable logistic regression analysis revealed that high postoperative FAR independently increased the occurrence of futile recanalization after adjusting for age, baseline NIHSS score, blood glucose at admission, occlusion site, and collateral status (aOR 2.40, 95%CI 1.18–4.87, *p* = .015; Figure 3). Heterogeneity in the association of high postoperative FAR with futile recanalization based on prior antithrombotic therapy, treatment of intravenous thrombolysis, occlusion site, time from symptom onset to groin puncture, and reperfusion status was insignificant (Figure 3). Sensitivity analysis using only patients with complete data obtained consistent results, with higher postoperative FAR levels being associated with futile recanalization (aOR 2.44, 95%CI 1.13–5.29, *p* = .023).

## 4 DISCUSSION

This study demonstrated an association between high postoperative FAR and futile recanalization in patients with acute anterior circulation large‐vessel occlusion and successful thrombectomy. The association was consistently observed regardless of treatment of intravenous thrombolysis, occlusion site, time from symptom onset to groin puncture, and reperfusion status.

Futile recanalization has been described as the failure to derive clinical benefit from a successful macrovascular recanalization (Ng et al., 2022). To date, the mechanisms of futile recanalization are poorly understood (Deng et al., 2022). No‐reflow phenomenon and inflammatory responses are thought to be important mechanisms (Deng et al., 2022; Wang & Xiong, 2023). The no‐reflow phenomenon is currently believed to reflect a compromise of the microvascular system, secondary to the dysfunction of endothelia and luminal clogging by leukocytes and microthrombi (Dalkara & Arsava, 2012; Ng et al., 2022). This combination could impair the perfusion of capillary beds and cause tissue damage despite an otherwise abundant blood supply from larger arteries (Ng et al., 2022). These processes could also accelerate the inflammatory cascade of both penumbral and infarcted tissue (Aly et al., 2021), which might eventually change blood hemorheology and cause inflammation. Supporting this theory, recent studies have found that futile recanalization can be predicted by postoperative hemostatic or inflammatory biomarkers, such as ADAMTS13 (a disintegrin and metalloproteinase with a thrombospondin type 1 motif, member 13) and high sensitivity C‐reactive protein (Feng et al., 2022; Zang et al., 2020).

Our results showed that a significantly higher postoperative FAR, potentially informing on both blood hemorheology and inflammation (Xiao et al., 2019), was observed in patients with futile recanalization compared with those without. Furthermore, a multivariable regression showed high postoperative FAR was associated with futile recanalization. The association maintained in subgroup analyses. However, the causative relationship between postoperative FAR and futile recanalization has yet to be proven. On the one hand, fibrinogen and albumin are acute‐phase reactants synthesized by the liver whose levels change positively and negatively, respectively (Beamer et al., 1993). It is possible that inflammatory responses in futile recanalization could shift protein synthesis to induce a higher FAR (Beamer et al., 1993). On the other hand, higher FAR can indicate a prothrombotic state resulting from alterations in hemostasis and blood viscosity due to higher levels of fibrinogen and/or lower levels of serum albumin (Acharya et al., 2020). This state could promote platelet aggregation and inhibit the physiological fibrinolytic system (Acharya et al., 2020; Zhao et al., 2019), which might induce microthrombi downstream and increase the likelihood of futile recanalization.

As the current rate of successful recanalization has reached approximately 90% due to tremendous advancements in thrombectomy technology, it is less likely that further improvement in devices will translate to a dramatic improvement in functional outcome (Desai et al., 2021). Cerebroprotection might be the next strategy to break through this ceiling (Desai et al., 2021). Our study supports the potential role that coagulation and inflammatory responses might play in the pathophysiology of futile recanalization. Neuroprotective agents with both anti‐inflammatory and anticoagulant effects, such as protease‐activated receptor‐1 agonist (3K3A‐activated protein C), may be targets of interest in future clinical trials (Ghozy et al., 2022; Lyden, 2021).

In addition, we observed that patients with low postoperative FAR more likely received treatment of intravenous thrombolysis. As thrombolytic agents have incomplete specificity to fibrin in thrombi, they could also cleave the circulating fibrinogen into fibrinogen degradation products (Matosevic et al., 2013). This process might decrease plasma fibrinogen levels and lower postoperative FAR after bridging therapy. Nevertheless, in subgroup and multiplicative interaction analyses, the association between high postoperative FAR with futile recanalization was maintained regardless of treatment with intravenous thrombolysis. History of receiving antithrombotic therapy, another potentially impactful factor of FAR, also did not influence this association, suggesting postoperative FAR can serve as a common indicator for futile recanalization.

This study has several limitations. First, it is a single‐center, retrospective study, which could inevitably cause selection bias. However, the results obtained seem both pathophysiologically plausible and clinically relevant. A prospective study is warranted to confirm the results obtained from this study. Second, fibrinogen and albumin could be affected by multiple factors, such as concurrent infections and malnutrition, which might influence its reliability as a prognostic marker for futile recanalization. However, the relative ease of obtaining this prognostic marker in a real‐world clinical setting might compensate for the above shortcoming. Third, preoperative FAR was not broadly obtained in our institution, which limited our analysis of the dynamic association between FAR and futile recanalization.

## 5 CONCLUSION

In conclusion, our findings support high postoperative FAR serving as an indicator of futile recanalization in patients with anterior circulation large‐vessel occlusion and successful thrombectomy.

## AUTHOR CONTRIBUTIONS

Di Li and Shen Li conceived and designed the study. Di Li, Tie‐Ping Fan, Xiao‐Yan Lan, Cong‐Jie Bi, Xu‐Sheng Zhao, Ming Mo, and Man‐Hong Zhao participated in data collection. Tao Tang and Lin‐Jia Guo did statistical analysis. Tao Tang drafted the manuscript. Tao Tang, Di Li, Tie‐Ping Fan, Johannes Boltze, Aline M. Thomas, Xun‐Ming Ji, and Shen Li critically revised the manuscript for important intellectual content. All authors provided final approval for the version of the manuscript submitted for publication and agree to be accountable for the work.

## CONFLICT OF INTEREST STATEMENT

The authors declare that there are no conflicts of interest.

### PEER REVIEW

The peer review history for this article is available at https://publons.com/publon/10.1002/brb3.3301.

## Data Availability

The data that support the findings of this study are available from the corresponding author upon reasonable request.

## References

[brb33301-bib-0001] Acharya, P. , Jakobleff, W. A. , Forest, S. J. , Chinnadurai, T. , Mellas, N. , Patel, S. R. , Kizer, J. R. , Billett, H. H. , Goldstein, D. J. , Jorde, U. P. , & Saeed, O. (2020). Fibrinogen albumin ratio and ischemic stroke during venoarterial extracorporeal membrane oxygenation. ASAIO Journal, 66(3), 277–282. 10.1097/MAT.0000000000000992 30973402 PMC7666805

[brb33301-bib-0002] Adams, H. P., Jr. , Bendixen, B. H. , Kappelle, L. J. , Biller, J. , Love, B. B. , Gordon, D. L. , & Marsh, E. E., 3rd (1993). Classification of subtype of acute ischemic stroke. Definitions for use in a multicenter clinical trial. TOAST. Trial of Org 10172 in Acute Stroke Treatment. Stroke; A Journal of Cerebral Circulation, 24(1), 35–41. 10.1161/01.str.24.1.35 7678184

[brb33301-bib-0003] Aly, M. , Abdalla, R. N. , Batra, A. , Shaibani, A. , Hurley, M. C. , Jahromi, B. S. , Potts, M. B. , & Ansari, S. A. (2021). Follow‐up neutrophil‐lymphocyte ratio after stroke thrombectomy is an independent biomarker of clinical outcome. Journal of NeuroInterventional Surgery, 13(7), 609–613. 10.1136/neurintsurg-2020-016342 32763917

[brb33301-bib-0004] Anadani, M. , Finitsis, S. , Clarencon, F. , Richard, S. , Marnat, G. , Bourcier, R. , Sibon, I. , Dargazanli, C. , Arquizan, C. , Blanc, R. , Lapergue, B. , Consoli, A. , Eugene, F. , Vannier, S. , Spelle, L. , Denier, C. , Boulanger, M. , Gauberti, M. , Liebeskind, D. S. , … ETIS Registry Investigators . (2022). Collateral status reperfusion and outcomes after endovascular therapy: Insight from the Endovascular Treatment in Ischemic Stroke (ETIS) registry. Journal of NeuroInterventional Surgery, 14(6), 551–557. 10.1136/neurintsurg-2021-017553 34140288

[brb33301-bib-0005] Austin, P. C. , White, I. R. , Lee, D. S. , & van Buuren, S. (2021). Missing data in clinical research: A tutorial on multiple imputation. Canadian Journal of Cardiology, 37(9), 1322–1331. 10.1016/j.cjca.2020.11.010 33276049 PMC8499698

[brb33301-bib-0006] Beamer, N. , Coull, B. M. , Sexton, G. , de Garmo, P. , Knox, R. , & Seaman, G. (1993). Fibrinogen and the albumin‐globulin ratio in recurrent stroke. Stroke; A Journal of Cerebral Circulation, 24(8), 1133–1139. 10.1161/01.str.24.8.1133 8342186

[brb33301-bib-0007] Collaborators, G. B. D. S. (2021). Global, regional, and national burden of stroke and its risk factors, 1990–2019: A systematic analysis for the global burden of disease study 2019. Lancet Neurology, 20(10), 795–820. 10.1016/S1474-4422(21)00252-0 34487721 PMC8443449

[brb33301-bib-0008] Dalkara, T. , & Arsava, E. M. (2012). Can restoring incomplete microcirculatory reperfusion improve stroke outcome after thrombolysis? Journal of Cerebral Blood Flow and Metabolism, 32(12), 2091–2099. 10.1038/jcbfm.2012.139 23047270 PMC3519416

[brb33301-bib-0009] Deng, G. , Xiao, J. , Yu, H. , Chen, M. , Shang, K. , Qin, C. , & Tian, D. S. (2022). Predictors of futile recanalization after endovascular treatment in acute ischemic stroke: A meta‐analysis. Journal of NeuroInterventional Surgery, 14(9), 881–885. 10.1136/neurintsurg-2021-017963 34544824

[brb33301-bib-0010] Desai, S. M. , Jha, R. M. , & Linfante, I. (2021). Collateral circulation augmentation and neuroprotection as adjuvant to mechanical thrombectomy in acute ischemic stroke. Neurology, 97(2), S178–S184. 10.1212/WNL.0000000000012809 34785616

[brb33301-bib-0011] Feng, Y. , Bai, X. , Li, W. , Cao, W. , Xu, X. , Yu, F. , Fu, Z. , Tian, Q. , Guo, X. , Wang, T. , Sha, A. , Chen, Y. , Gao, P. , Wang, Y. , Chen, J. , Ma, Y. , Chen, F. , Dmytriw, A. A. , Regenhardt, R. W. , … Jiao, L. (2022). Postoperative neutrophil‐lymphocyte ratio predicts unfavorable outcome of acute ischemic stroke patients who achieve complete reperfusion after thrombectomy. Frontiers in immunology, 13, 963111. 10.3389/fimmu.2022.963111 36275640 PMC9585914

[brb33301-bib-0012] Ghozy, S. , Reda, A. , Varney, J. , Elhawary, A. S. , Shah, J. , Murry, K. , Sobeeh, M. G. , Nayak, S. S. , Azzam, A. Y. , Brinjikji, W. , Kadirvel, R. , & Kallmes, D. F. (2022). Neuroprotection in acute ischemic stroke: A battle against the biology of nature. Frontiers in Neurology, 13, 870141. 10.3389/fneur.2022.870141 35711268 PMC9195142

[brb33301-bib-0013] Goyal, M. , Menon, B. K. , van Zwam, W. H. , Dippel, D. W. , Mitchell, P. J. , Demchuk, A. M. , Dávalos, A. , Majoie, C. B. , van der Lugt, A. , de Miquel, M. A. , Donnan, G. A. , Roos, Y. B. , Bonafe, A. , Jahan, R. , Diener, H. C. , van den Berg, L. A. , Levy, E. I. , Berkhemer, O. A. , Pereira, V. M. , … collaborators, H. (2016). Endovascular thrombectomy after large‐vessel ischaemic stroke: A meta‐analysis of individual patient data from five randomised trials. Lancet, 387(10029), 1723–1731. 10.1016/S0140-6736(16)00163-X 26898852

[brb33301-bib-0014] Kim, B. J. , Singh, N. , & Menon, B. K. (2021). Hemodynamics of leptomeningeal collaterals after large vessel occlusion and blood pressure management with endovascular treatment. Journal of Stroke, 23(3), 343–357. 10.5853/jos.2021.02446 34649379 PMC8521259

[brb33301-bib-0015] Lyden, P. D. (2021). Cerebroprotection for acute ischemic stroke: Looking ahead. Stroke; A Journal of Cerebral Circulation, 52(9), 3033–3044. 10.1161/STROKEAHA.121.032241 PMC8384682 34289710

[brb33301-bib-0016] Matosevic, B. , Knoflach, M. , Werner, P. , Pechlaner, R. , Zangerle, A. , Ruecker, M. , Kirchmayr, M. , Willeit, J. , & Kiechl, S. (2013). Fibrinogen degradation coagulopathy and bleeding complications after stroke thrombolysis. Neurology, 80(13), 1216–1224. 10.1212/WNL.0b013e3182897015 23486872

[brb33301-bib-0017] Ng, F. C. , Churilov, L. , Yassi, N. , Kleinig, T. J. , Thijs, V. , Wu, T. , Shah, D. , Dewey, H. , Sharma, G. , Desmond, P. , Yan, B. , Parsons, M. , Donnan, G. , Davis, S. , Mitchell, P. , & Campbell, B. (2022). Prevalence and significance of impaired microvascular tissue reperfusion despite macrovascular angiographic reperfusion (No‐Reflow). Neurology, 98(8), e790–e801. 10.1212/WNL.0000000000013210 34906976

[brb33301-bib-0018] Nie, X. , Leng, X. , Miao, Z. , Fisher, M. , & Liu, L. (2023). Clinically ineffective reperfusion after endovascular therapy in acute ischemic stroke. Stroke; A Journal of Cerebral Circulation, 54(3), 873–881. 10.1161/STROKEAHA.122.038466 36475464

[brb33301-bib-0019] Powers, W. J. , Rabinstein, A. A. , Ackerson, T. , Adeoye, O. M. , Bambakidis, N. C. , Becker, K. , Biller, J. , Brown, M. , Demaerschalk, B. M. , Hoh, B. , Jauch, E. C. , Kidwell, C. S. , Leslie‐Mazwi, T. M. , Ovbiagele, B. , Scott, P. A. , Sheth, K. N. , Southerland, A. M. , Summers, D. V. , Tirschwell, D. L. , & Tirschwell, D. L. (2019). Guidelines for the early management of patients with acute ischemic stroke: 2019 update to the 2018 guidelines for the early management of acute ischemic stroke: A guideline for healthcare professionals from the American Heart Association/American Stroke Association. Stroke; A Journal of Cerebral Circulation, 50(12), e344–e418. 10.1161/STR.0000000000000211 31662037

[brb33301-bib-0020] Roth, S. , Jansen, C. , M'Pembele, R. , Stroda, A. , Boeken, U. , Akhyari, P. , Lichtenberg, A. , Hollmann, M. W. , Huhn, R. , Lurati Buse, G. , & Aubin, H. (2021). Fibrinogen‐albumin‐ratio is an independent predictor of thromboembolic complications in patients undergoing VA‐ECMO. Scientific Reports, 11(1), 16648. 10.1038/s41598-021-95689-x 34404824 PMC8371004

[brb33301-bib-0021] Ruan, Y. , Yuan, C. , Liu, Y. , Zeng, Y. , Cheng, H. , Cheng, Q. , Chen, Y. , Huang, G. , He, W. , & He, J. (2021). High fibrinogen‐to‐albumin ratio is associated with hemorrhagic transformation in acute ischemic stroke patients. Brain and Behavior, 11(1), e01855. 10.1002/brb3.1855 33314645 PMC7821560

[brb33301-bib-0022] Sang, H. , Xie, D. , Tian, Y. , Nguyen, T. N. , Saver, J. L. , Nogueira, R. G. , Wu, J. , Long, C. , Tian, Z. , Hu, Z. , Wang, T. , Li, R. , Ke, Y. , Zhu, X. , Peng, D. , Chang, M. , Li, L. , Ruan, J. , Wu, D. , … Qiu, Z. (2023). Association of tirofiban with functional outcomes after thrombectomy in acute ischemic stroke due to intracranial atherosclerotic disease. Neurology, 100(19), e1996–e2006. 10.1212/WNL.0000000000207194 36941074 PMC10186214

[brb33301-bib-0023] Tu, W. J. , & Wang, L. D. , & Special Writing Group of China Stroke Surveillance, R . (2023). China stroke surveillance report 2021. Military Medical Research, 10(1), 33. 10.1186/s40779-023-00463-x 37468952 PMC10355019

[brb33301-bib-0024] Tu, W. J. , Zhao, Z. , Yin, P. , Cao, L. , Zeng, J. , Chen, H. , Fan, D. , Fang, Q. , Gao, P. , Gu, Y. , Tan, G. , Han, J. , He, L. , Hu, B. , Hua, Y. , Kang, D. , Li, H. , Liu, J. , Liu, Y. , … Wang, L. (2023). Estimated burden of stroke in China in 2020. JAMA Network Open, 6(3), e231455. 10.1001/jamanetworkopen.2023.1455 36862407 PMC9982699

[brb33301-bib-0025] Wang, L. , & Xiong, Y. (2023). Advances in futile reperfusion following endovascular treatment in acute ischemic stroke due to large vessel occlusion. European Neurology, 86(2), 95–106. 10.1159/000528922 36754030

[brb33301-bib-0026] Xiao, L. , Jia, Y. , Wang, X. , & Huang, H. (2019). The impact of preoperative fibrinogen‐albumin ratio on mortality in patients with acute ST‐segment elevation myocardial infarction undergoing primary percutaneous coronary intervention. Clinica Chimica Acta, 493, 8–13. 10.1016/j.cca.2019.02.018 30796900

[brb33301-bib-0027] Xu, Y. , Liu, C. , Li, W. , Nie, X. , Huang, S. , Li, X. , Wu, Y. , Jin, W. S. , Jiang, J. , Dong, J. , Yang, Y. , Sun, Z. , Han, W. , Wang, Y. , Liu, L. , & Zhang, M. (2023). Efficacy and safety of early anticoagulation after endovascular treatment in patients with atrial fibrillation. Stroke and Vascular Neurology, 8, 405–412. 10.1136/svn-2022-002082 36972921 PMC10647876

[brb33301-bib-0028] Yang, X. , Sun, D. , Huo, X. , Raynald, R. , Jia, B. , Tong, X. , Wang, A. , Ma, N. , Gao, F. , Mo, D. , & Miao, Z. , & ANGEL‐ACT study group . (2023). Futile reperfusion of endovascular treatment for acute anterior circulation large vessel occlusion in the ANGEL‐ACT registry. Journal of NeuroInterventional Surgery. 10.1136/jnis-2022-019874 36693725

[brb33301-bib-0029] Zaidat, O. O. , Yoo, A. J. , Khatri, P. , Tomsick, T. A. , von Kummer, R. , Saver, J. L. , Marks, M. P. , Prabhakaran, S. , Kallmes, D. F. , Fitzsimmons, B. F. , Mocco, J. , Wardlaw, J. M. , Barnwell, S. L. , Jovin, T. G. , Linfante, I. , Siddiqui, A. H. , Alexander, M. J. , Hirsch, J. A. , Wintermark, M. , … STIR Thrombolysis in Cerebral Infarction (TICI) Task Force . (2013). Recommendations on angiographic revascularization grading standards for acute ischemic stroke: A consensus statement. Stroke; A Journal of Cerebral Circulation, 44(9), 2650–2663. 10.1161/STROKEAHA.113.001972 PMC4160883 23920012

[brb33301-bib-0030] Zang, N. , Lin, Z. , Huang, K. , Pan, Y. , Wu, Y. , Wu, Y. , & Pan, S. (2020). Biomarkers of unfavorable outcome in acute ischemic stroke patients with successful recanalization by endovascular thrombectomy. Cerebrovascular Diseases, 49(6), 583–592. 10.1159/000510804 33105129

[brb33301-bib-0031] Zhao, Y. , Yang, J. , Ji, Y. , Wang, S. , Wang, T. , Wang, F. , & Tang, J. (2019). Usefulness of fibrinogen‐to‐albumin ratio to predict no‐reflow and short‐term prognosis in patients with ST‐segment elevation myocardial infarction undergoing primary percutaneous coronary intervention. Heart and Vessels, 34(10), 1600–1607. 10.1007/s00380-019-01399-w 30993442

